# Diagnostic Performance of Salusins in Acute Pulmonary Embolism: A Prospective Observational Study

**DOI:** 10.3390/diagnostics15162105

**Published:** 2025-08-21

**Authors:** Tuğba Sanalp Menekşe, İlker Şirin, Rabia Handan Günsay, Uğurcan Eker, Rasime Pelin Kavak, Yavuz Otal, Canan Topçuoğlu

**Affiliations:** 1 Department of Emergency Medicine, Ankara Etlik City Hospital, 06170 Ankara, Turkey; sirinilkerr@gmail.com (İ.Ş.); dr.handan.karaatli@gmail.com (R.H.G.); dryotal@gmail.com (Y.O.); 2 Department of Medical Biochemistry, Ankara Etlik City Hospital, 06170 Ankara, Turkey; ueker2053@gmail.com (U.E.); drcanantopcuoglu@gmail.com (C.T.); 3 Department of Radiology, Ankara Etlik City Hospital, 06170 Ankara, Turkey; drrpelindemir6@hotmail.com

**Keywords:** biomarkers, computed tomography angiography, emergency service, hospital, prognosis, pulmonary embolism, salusin-α

## Abstract

**Background/Objectives**: This study investigated whether serum salusin-α and salusin-β levels could support the diagnosis and prognosis of confirmed acute pulmonary embolism (APE) cases. **Methods**: A prospective observational study was conducted including 57 patients diagnosed with APE using computed tomography pulmonary angiography (CTPA) and 30 control participants without any acute or chronic disease. APE patients were categorized based on the Pulmonary Artery Obstruction Index (PAOI) into low (≤20) and high (>20) thrombus burden groups. Serum salusin-α and salusin-β levels were measured at diagnosis using an enzyme-linked immunosorbent assay. Associations with PAOI and Pulmonary Embolism Severity Index (PESI) scores were analyzed. **Results**: Salusin-α and salusin-β levels were markedly reduced in APE patients versus controls (*p* < 0.001). In multivariate analysis, salusin-α remained independently associated with APE (*p* = 0.042), whereas salusin-β was not significant. A receiver operating characteristic analysis showed good diagnostic performance for salusin-α (AUC = 0.799; sensitivity = 89.5%; specificity = 46.7%). Neither peptide correlated with PAOI or PESI. At a 305.85 pg/mL cut-off, salusin-α yielded a positive predictive value of 76.1% and a negative predictive value of 70% in this cohort. **Conclusions**: The findings suggest that salusin-α has high sensitivity in detecting acute pulmonary embolism and may serve as a supportive diagnostic marker in emergency settings. Although its specificity is limited, it could contribute to guiding additional testing. While salusin-β showed no significant diagnostic value, the potential role of salusin peptides in prognostic evaluation requires further exploration.

## 1. Introduction

Acute pulmonary embolism (APE) represents a critical cardiovascular emergency with potential for sudden-onset symptoms and significant hemodynamic instability [1]. In the general population, APE has an annual incidence ranging from 40 to 110 cases per 100,000 individuals. It accounts for approximately between 0.2% and 0.3% of emergency department (ED) visits and carries a mortality risk of over 20% within the first 30 days, particularly in high-risk patients [2,3]. The variability in clinical presentation, the lack of specific symptoms, and the fact that some cases may remain asymptomatic make early diagnosis challenging. Furthermore, the absence of reliable prognostic markers complicates patient management [4]. Therefore, especially in ED settings, there remains a pressing need for novel and dependable parameters that can aid in diagnosis and help predict the clinical course of the disease [5,6].

One of the most commonly used biomarkers in the diagnosis of APE is D-dimer, which has high sensitivity due to its ability to indicate fibrin degradation products. It is particularly valuable as a clinical exclusion tool in patients with low to intermediate risk [7]. In cases where D-dimer levels are elevated or when APE is clinically suspected, computed tomography pulmonary angiography (CTPA), which provides direct visualization of the pulmonary arteries with high anatomical resolution and specificity, is considered the reference imaging modality [8,9]. However, D-dimer levels may also be physiologically elevated in various clinical conditions such as advanced age, inflammatory states, or certain comorbidities. This reduces the test’s specificity and increases the rate of false-positive results [10]. Consequently, this may lead to unnecessary imaging requests, delays in the diagnostic process, and additional financial burdens on the healthcare system [11]. Although CTPA offers high diagnostic accuracy, it is not feasible for routine use in every patient due to factors such as limited accessibility, cost, and exposure to ionizing radiation. For all these reasons, the development of new biomarkers that are both diagnostically accurate and practically applicable, as well as specific and reliable, remains an important clinical need in patients with APE [12].

Salusin peptides are endogenously produced, biologically active molecules derived from a precursor protein known as prosalusin, and they exist in two isoforms: salusin-alpha (α) and salusin-beta (β) [13]. Salusin-α has been shown to inhibit atherosclerotic progression by preventing lipid accumulation in the vascular wall, whereas salusin-β appears to accelerate this process. These peptides are thought to play roles in several fundamental steps of atherogenesis, including endothelial injury, proliferation of vascular smooth muscle cells, activation of inflammatory responses, and transformation of macrophages into foam cells [14]. Moreover, growing evidence suggests that salusins may be involved not only in cardiovascular diseases but also in the pathogenesis and clinical progression of various systemic disorders [15,16,17]. Given their broad spectrum of actions at the cellular and molecular levels, salusins have emerged as promising biomarker candidates with potential applications in both diagnosis and prognosis.

Considering the thromboembolic processes and vascular inflammation mechanisms involved in the pathogenesis of APE, salusin peptides may be potential candidate biomarkers for this condition. However, diagnostic data regarding salusin levels in patients with APE remain limited. In this study, serum salusin-α and salusin-β levels were compared between patients diagnosed with APE in the ED and healthy controls in order to evaluate the diagnostic utility and potential prognostic significance of these peptides.

## 2. Materials and Methods

### 2.1. Study Design and Setting

The study was conducted at the Emergency Medicine Department of Ankara Etlik City Hospital as a prospective observational cohort. Ethical approval was obtained from the institution’s Clinical Research Ethics Committee (Approval No: AEŞK-BADEK-2025-0573). Following ethical approval, patient recruitment began on 27 March 2025, and the data collection process was completed by the end of May 2025, lasting approximately two months. Written informed consent was obtained from all participants. All data were anonymized in accordance with principles of confidentiality and used solely for scientific purposes. The study was conducted in accordance with the ethical principles outlined in the Declaration of Helsinki.

### 2.2. Patient Selection and Eligibility Criteria

Adult patients (≥18 years) who presented to the ED and were diagnosed with APE based on CTPA findings were enrolled, following the 2019 guidelines of the European Society of Cardiology [18]. All participants provided written informed consent prior to inclusion. The exclusion criteria included: patients with chronic pulmonary embolism; patients transferred from other healthcare centers after receiving initial treatment; those with an active malignancy or recent (within six months) history of oncological therapy; individuals with chronic or end-stage renal disease; a known history of acute or chronic trauma; recent major surgery (within three months); autoimmune diseases with chronic inflammation, particularly lupus or rheumatoid arthritis; pregnancy, the postpartum period, or lactation; signs of severe sepsis or systemic infection; inability to obtain a high-quality blood sample for biomarker evaluation; and patients who refused to participate or voluntarily withdrew during the study.

Among 71 patients who met the study criteria and had a confirmed diagnosis of APE via CTPA, 57 were included in the study through simple random sampling using Microsoft Excel and R software (version 4.4.3, R Foundation for Statistical Computing, Vienna, Austria). During the same period, 30 individuals who presented to the ED with non-specific symptoms but were not diagnosed with any acute or chronic illness—and had no history of thromboembolic disease—were randomly selected to form the control group. Both groups were matched for age and sex, and no pathological findings were detected in the laboratory evaluations of the control participants. To evaluate their potential clinical relevance, salusin-α and salusin-β levels were determined from blood samples collected during the routine diagnostic process. No additional diagnostic or therapeutic interventions were performed on any participant.

### 2.3. Sample Size Calculation

Prior to data collection, a sample size calculation was carried out using G*Power version 3.1 to ensure adequate statistical power. Assuming a medium effect size (Cohen’s f = 0.5), an alpha level of 0.05, and a desired power of 98%, the analysis indicated that a minimum of 81 participants (27 per group) would be needed to detect clinically meaningful differences across the three groups: healthy controls, patients with low thrombus burden, and those with high thrombus burden [19]. Ultimately, the final sample met the minimum calculated size, with group sizes being kept as balanced as possible based on the availability of 87 salusin assay kits.

### 2.4. Radiological Evaluation—Calculation of the Pulmonary Artery Obstruction Index (PAOI)

In cases diagnosed with pulmonary embolism, the PAOI, as described by Qanadli et al., was used to quantitatively assess embolic burden [20]. This method provides a percentage-based representation of vascular obstruction based on the location of the embolus and the degree of occlusion visualized through CTPA. Assuming that each lung contains ten segmental arteries, the product of the number of affected segments (*n*) and the degree of obstruction (d) is calculated. The parameter d was coded as 1 to indicate partial occlusion and as 2 to represent complete occlusion. To express the degree of obstruction as a percentage, the cumulative score was first divided by the theoretical maximum of 40 (derived from 20 segments with a maximum weight of 2), and the result was then multiplied by 100. The following formula represents this calculation:

PAOI (%) = [Σ(*n* × d)/40] × 100

**Figure 1:**
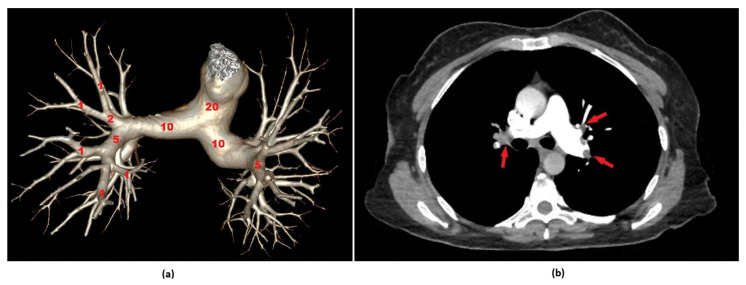
Representative computed tomography pulmonary angiography (CTPA) images illustrating Qanadli scoring and Pulmonary Artery Obstruction Index (PAOI) calculation. (**a**) A three-dimensional volume-rendered reconstruction of the pulmonary arterial tree based on CTPA, illustrating the principles of Qanadli’s scoring system. Each pulmonary segment is assessed for the presence and extent of embolic obstruction. Segmental arteries are assigned 1 point for partial and 2 points for complete occlusion, while lobar and main pulmonary arteries contribute proportionally based on involved branches. The cumulative score, which has a maximum value of 40, is used to calculate the PAOI, yielding a percentage-based quantification of embolic burden. (**b**) Axial CTPA image of a 48-year-old female patient who presented to the emergency department with dyspnea. A partial occlusion is observed in the left interlobar pulmonary artery and a complete occlusion in the right interlobar artery (red arrows). The Qanadli score was calculated as 29, corresponding to a PAOI of 72.5%.

The radiological evaluations were performed on a dedicated workstation equipped with multiplanar reconstruction capabilities and mediastinal window settings. All imaging analyses were independently conducted by a radiologist (R.P.K.) with 17 years of professional experience in thoracic imaging, who was blinded to the patients’ clinical information throughout the assessment process. In this study, thrombus burden was classified based on the PAOI value. Using a threshold of 20 points, which is commonly referenced in the literature, patients with a PAOI of 20 or below were defined as having a low thrombus burden, while those with a score above 20 were considered to have a high thrombus burden [20]. Representative computed tomography images illustrating Qanadli scoring and PAOI calculation are presented in Figure 1a,b.

### 2.5. Clinical Risk Stratification Using the Pulmonary Embolism Severity Index (PESI)

The PESI is a validated scoring system developed to classify clinical risk and predict short-term mortality in patients diagnosed with APE [18]. It incorporates both demographic and clinical variables, including age, sex, history of cancer, chronic cardiovascular or pulmonary conditions, vital signs such as systolic blood pressure (SBP), respiratory and heart rates, body temperature, mental status, and oxygen saturation. Based on the overall score, patients are classified into five risk categories, where higher scores are associated with an elevated likelihood of mortality within 30 days.

### 2.6. Outcome Measures

The primary outcome investigated the association between serum salusin-α and salusin-β levels and the presence of APE, as well as the ability of these biomarkers to distinguish between patients with low and high thrombus burden, as classified by the PAOI. Additionally, the relationship between salusin levels and clinical severity was evaluated using the PESI score.

Secondary outcomes assessed in the ED setting included 30-day clinical endpoints such as the need for intensive care admission, administration of thrombolytic therapy, hospital length of stay, and all-cause mortality.

### 2.7. Analysis of Biochemical Parameters

Venous blood samples of 5 mL were drawn from each participant into serum separator tubes. The samples were centrifuged at 3000× *g* for 15 min within 30 to 60 min of collection to isolate serum. N-terminal pro-B-type natriuretic peptide (NT-proBNP) and high-sensitivity troponin T (hs-TnT) levels were measured using a Cobas 8000 modular analyzer (Roche Diagnostics GmbH, Mannheim, Germany). Both biomarkers were analyzed using the electrochemiluminescence immunoassay method. D-dimer levels were analyzed using a Cobas t711 coagulation analyzer (Roche Diagnostics GmbH, Mannheim, Germany), based on the latex-enhanced immunoturbidimetric assay principle. These analyses were performed on the same day the blood was drawn. For salusin-α and salusin-β analysis, serum samples were aliquoted into Eppendorf tubes immediately after centrifugation and stored at −80 °C until analysis. Samples showing hemolysis were excluded from the analysis.

### 2.8. Measurement and Evaluation of Salusin Peptides in Serum Samples

Serum concentrations of salusin-α and salusin-β were determined by an enzyme-linked immunosorbent assay utilizing commercially available kits (BT Lab, Biotechnology Laboratory, Shanghai, China; Catalog No: E1268Hu for Salusin-α, Catalog No: E1272Hu for Salusin-β), following the manufacturer’s protocol. The measurement range for salusin-α was 5–1000 pg/mL, with a sensitivity of 0.51 pg/mL. For salusin-β, the measurement range was 10–1800 pg/mL, with a sensitivity of 5.22 pg/mL. Both kits demonstrated intra-assay and inter-assay coefficients of variation below 8% and 10%, respectively. Absorbance measurements were conducted at 450 nm with a microplate reader.

### 2.9. Statistical Analysis

Statistical analyses were conducted using IBM SPSS Statistics for Windows, version 26.0 (IBM Corp., Armonk, NY, USA). The distribution normality of continuous variables was assessed via the Kolmogorov–Smirnov test. Normally distributed data are expressed as the mean ± standard deviation, while non-normal data are presented as the median with the interquartile range. Categorical data are summarized as counts and percentages. Between-group comparisons for normally distributed variables were performed using the independent samples *t*-test, whereas the Mann–Whitney U test was applied for non-normally distributed variables. Categorical variables were compared using the chi-square test or Fisher’s exact test as appropriate. A Spearman correlation analysis was employed to evaluate the associations of salusin-α and salusin-β levels with PESI scores and PAOI. The diagnostic and prognostic performance of salusin-α and salusin-β was examined through a receiver operating characteristic (ROC) curve analysis, with the area under the curve (AUC) values calculated. The Youden index was utilized to determine optimal cut-off values, balancing sensitivity and specificity. To explore factors independently associated with APE, logistic regression models were applied. Both univariate and multivariate analyses were performed, adjusting for key variables such as sex, age, PESI score, and PAOI. Statistical significance was set at *p* < 0.05 for all analyses.

## 3. Results

**Figure 2:**
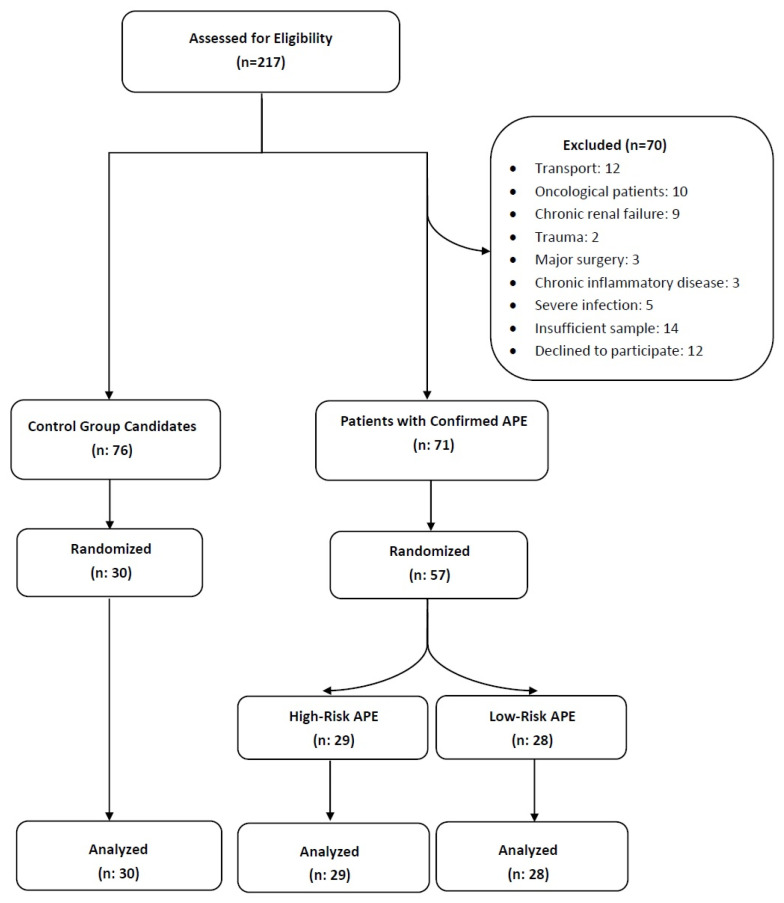
Flowchart summarizing the exclusion criteria and the final study population. Abbreviations: APE, acute pulmonary embolism.

After excluding individuals who met the exclusion criteria, a total of 87 participants were included in the final analysis. Of these, 57 were patients diagnosed with APE and 30 were non-diseased individuals serving as controls. The patient selection process is summarized in the flowchart presented in Figure 2.

**Figure 3:**
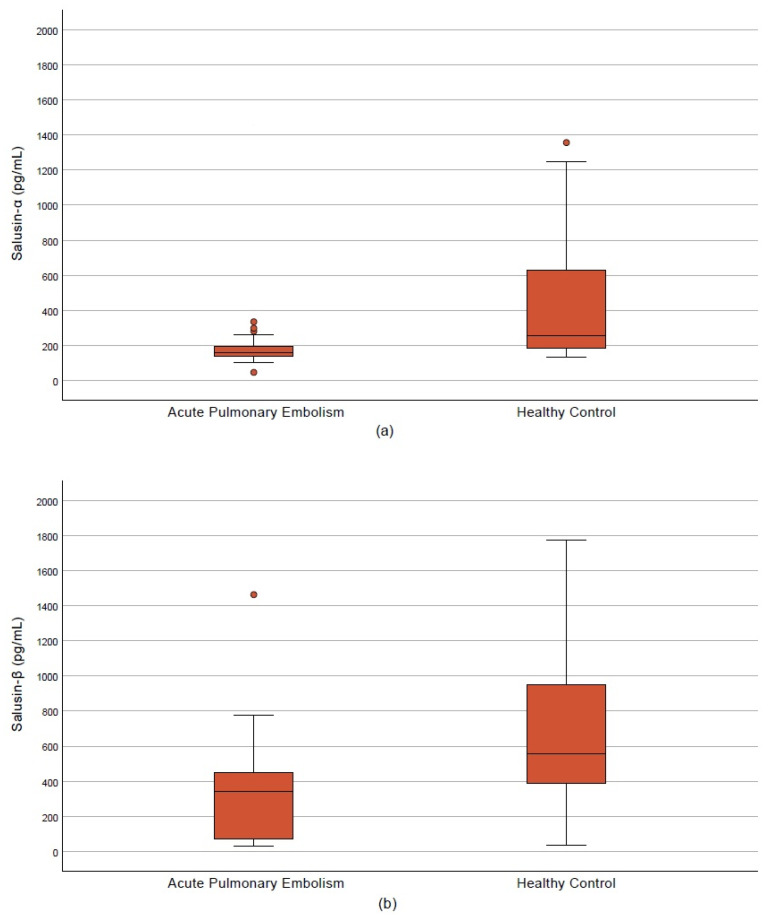
Salusin levels in the acute pulmonary embolism and control groups. (**a**) Salusin-α. (**b**) Salusin-β.

The mean age was significantly higher in the APE group compared with controls (61.96 ± 19.30 vs. 37.83 ± 8.35 years, *p* < 0.001). No significant difference was observed in sex distribution (50.9% vs. 46.7% male, *p* = 0.709). BMI was significantly elevated in patients with APE (*p* < 0.001). Systolic and diastolic blood pressures, along with SpO_2_ levels, were lower, while respiratory and heart rates were higher in the APE group (*p* < 0.05). Cardiac biomarkers including hs-TnT, D-dimer, and NT-proBNP were significantly increased (*p* < 0.001) (Table 1). Serum salusin-α and salusin-β levels were significantly reduced in APE patients compared with controls (159.5 vs. 257.15 pg/mL and 341.7 vs. 555.85 pg/mL, respectively; *p* < 0.001) (Table 1, Figure 3a,b).

High-risk patients (*n* = 29) were significantly older and had a different sex distribution compared with low-risk patients (*n* = 28) (*p* = 0.007 and *p* = 0.047, respectively). The high-risk group showed lower SpO_2_ levels (*p* = 0.019), elevated hs-TnT and D-dimer levels (*p* = 0.001 and *p* < 0.001), and higher PESI scores (*p* = 0.002). Clinically, these patients more frequently required intensive care (*p* = 0.002) and thrombolytic therapy (*p* = 0.023). No significant differences were observed between the groups regarding salusin-α and salusin-β levels (*p* = 0.307 and *p* = 0.854) (Table 2).

Univariate regression analysis revealed a significant negative association between salusin-α levels and APE (OR = 0.996; 95% CI: 0.994–0.999; *p* = 0.030), which persisted after adjusting for age, BMI, and SBP in the multivariate model (OR = 0.995; 95% CI: 0.990–1.000; *p* = 0.042) (Table 3). Salusin-β was significant in univariate analysis (OR = 0.998; 95% CI: 0.996–0.999; *p* = 0.020) but lost significance in the multivariate model (*p* = 0.088) (Table 4). Age and BMI showed positive associations with APE, whereas SBP was inversely related.

**Figure 4:**
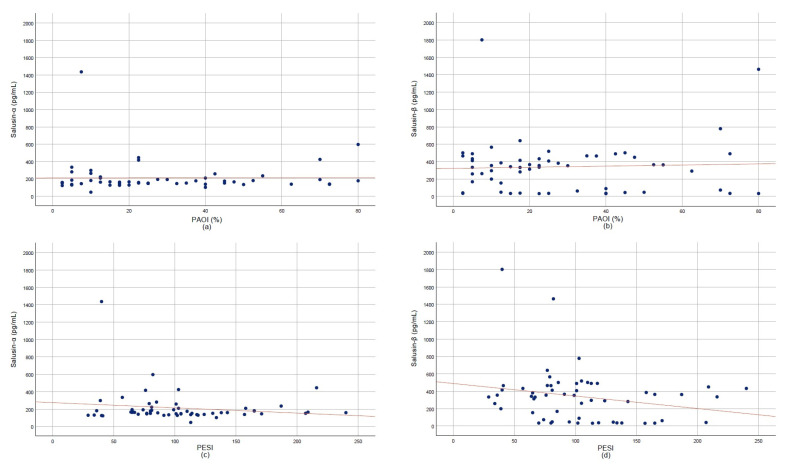
Scatter plots showing correlations of salusin-α and salusin-β with clinical indices. (**a**) Pulmonary Artery Obstruction Index (PAOI) vs. salusin-α. (**b**) PAOI vs. salusin-β. (**c**) Pulmonary Embolism Severity Index (PESI) vs. salusin-α. (**d**) PESI vs. salusin-β.

**Figure 5:**
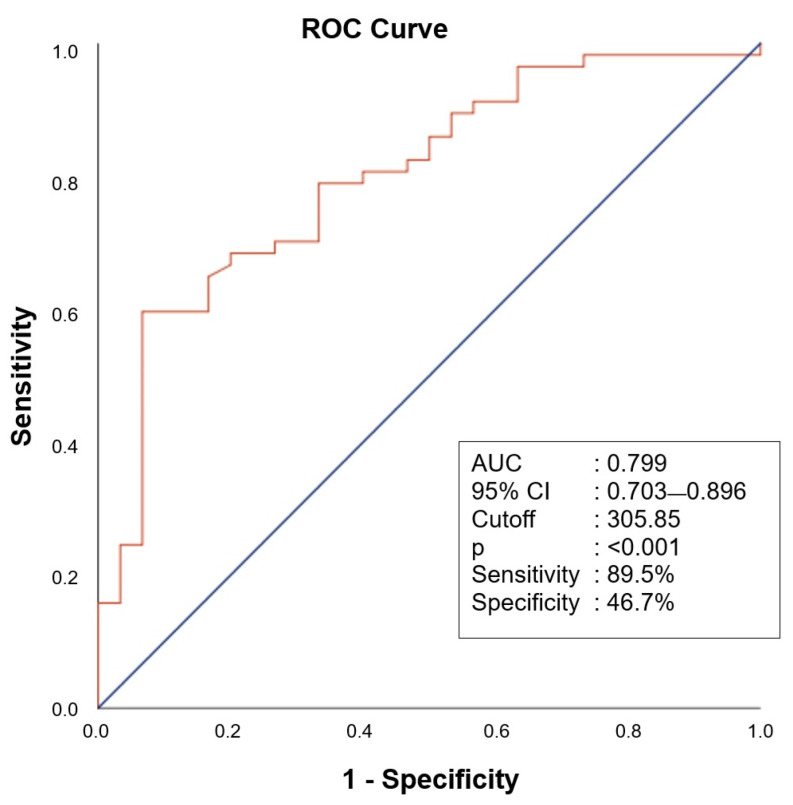
Receiver operating characteristic curve analysis of salusin-α for values below 305.85 in predicting acute pulmonary embolism.

Correlation analyses indicated weak, non-significant trends between both salusin isoforms and PAOI (Figure 4a,b) as well as PESI scores (Figure 4c,d) (*p* > 0.05). The ROC curve analysis demonstrated that salusin-α had moderate diagnostic accuracy with an AUC of 0.799 (95% CI: 0.703–0.896). The diagnostic performance of salusin-α was evaluated using the ROC curve analysis, and the AUC was found to be 0.799 (95% CI: 0.703–0.896). At a cut-off value of 305.85 pg/mL, the sensitivity and specificity were calculated as 89.5% and 46.7%, respectively. These findings suggest that salusin-α may be a potential biomarker with significant discriminatory power for the diagnosis of APE (*p* < 0.001) (Figure 5).

## 4. Discussion

### 4.1. Role of Salusin-α and Salusin-β as Diagnostic Biomarkers

In this prospective observational study, serum levels of salusin-α and salusin-β were significantly reduced in patients diagnosed with APE compared with controls without acute or chronic illness. Notably, salusin-α demonstrated a statistically significant association with the presence of APE in both univariate and multivariate analyses. The area under the AUC for salusin-α was calculated as 0.799, with a sensitivity of 89.5%, indicating its potential as a biomarker with meaningful discriminatory power in the diagnosis of APE. In contrast, although salusin-β was significant in univariate analysis, it lost statistical significance in the multivariate model. As far as we are aware, no previous research has examined salusin peptide levels in patients with APE, making our results a novel addition to the literature.

The non-specific nature of APE symptoms often complicates early and accurate diagnosis, particularly in ED settings. This challenge underscores the need for reliable biomarkers that can support clinical decision-making [21,22]. Although D-dimer testing is frequently used in this context, its low specificity in patients with low to intermediate risk has been widely reported to cause false-positive results, leading to unnecessary advanced imaging studies [12,23]. For example, in a study by Sekar et al., the specificity of the D-dimer test was reported as only 20%, with a positive predictive value of 32% [12]. These findings indicate that approximately 68% of patients with positive D-dimer results do not actually have APE, contributing to widespread overuse of imaging resources. In the present study, salusin-α emerged as a significant marker in multivariate analysis and demonstrated higher specificity compared with D-dimer. This finding suggests that salusin-α may serve as a complementary tool in the diagnostic process, potentially improving the efficiency of clinical decision-making. Moreover, the high sensitivity and AUC value observed in the ROC analysis indicate that salusin-α holds notable potential for early diagnosis. Particularly in cases where D-dimer levels are elevated but clinical assessment suggests a low probability of embolism, incorporating salusin-α measurements may enable more selective use of imaging and help reduce unnecessary CTPA procedures.

### 4.2. Potential Pathophysiological Role of Salusins in APE

Salusin peptides are known to exert dual effects on vascular tone, inflammation, and cellular proliferation [24]. The endothelial-protective and anti-inflammatory properties of salusin-α suggest that it may serve as a potential biomarker in clinical conditions associated with vascular injury and thrombosis. In contrast, the pro-inflammatory effects of salusin-β indicate that it may be involved in different pathophysiological processes. From this perspective, salusins are considered critical regulatory molecules that influence cardiovascular homeostasis [25]. Given the pathophysiology of APE, which involves vascular endothelial injury, activation of inflammatory pathways, and a pronounced procoagulant response [26], the vascular-stabilizing properties of salusin-α—such as supporting endothelial function, suppressing inflammation, and limiting platelet activity—suggest that this peptide may play a protective role in thromboinflammatory conditions like APE [27]. The low salusin-α levels observed in APE patients in our study may reflect this underlying biological response. Indeed, previous studies have reported reduced salusin-α levels in association with cardiovascular diseases and systemic inflammatory conditions [28,29,30]. Therefore, salusin-α may be considered not only a diagnostic biomarker but also a promising tool for gaining insight into the underlying pathophysiology of the disease.

Recent evaluations have shown that salusin-β exerts significant effects on vascular endothelial integrity and may contribute to the development of vascular diseases such as hypertension and atherosclerosis, primarily by amplifying inflammatory responses [31]. In a prospective study conducted by Janecka and Stefanowicz in 2024, serum salusin-β levels were reported to be positively correlated with atherosclerosis and components of metabolic syndrome [25]. These findings suggest that salusin-β may play a role in processes such as vascular wall inflammation and endothelial dysfunction. However, although these mechanisms theoretically overlap with thromboinflammatory processes like those seen in APE, the absence of a significant diagnostic difference in salusin-β levels in our study is noteworthy. This may indicate that salusin-β is more involved in chronic inflammatory and metabolic processes rather than in the early-stage pathophysiology of APE. Therefore, in order to clarify the potential diagnostic value of salusin-β in APE, further advanced clinical studies are needed, taking into account its time-dependent and tissue-specific effects.

### 4.3. Association of Salusins with Thrombus Burden and Clinical Risk Scores

The PESI is a widely used tool for predicting short-term mortality risk in patients with APE based on clinical parameters, while the PAOI is an anatomical scoring system that quantitatively assesses thrombus burden using imaging findings [32,33]. Previous studies have reported that markers such as hs-TnT, NT-proBNP, D-dimer, and various inflammatory indicators show correlations with these clinical scoring systems, contributing to both diagnosis and prognostication [34,35]. However, there is a lack of sufficient data in the literature regarding the relationship between salusin peptides and these scoring systems.

In our study, salusin-α and salusin-β levels were not found to be statistically correlated with either PAOI or PESI scores. Similarly, subgroup analyses comparing low-, intermediate-, and high-risk categories revealed no significant differences in salusin levels. These findings suggest that salusins may not be directly associated with the anatomical extent or clinical severity of APE. Possible explanations for this observation include the role of salusins in early inflammatory responses rather than in later clinical manifestations, lack of association with advanced clinical indicators, inter-individual biological variability, limited sample size, and fluctuations in salusin levels during the acute phase.

### 4.4. Prognostic Reflections of Salusin Levels on Clinical Outcomes

Studies evaluating the potential relationship between salusin peptides and prognostic parameters are currently quite limited. The existing literature has primarily focused on salusin levels in the context of inflammation and endothelial dysfunction in cardiovascular diseases [15], whereas studies directly examining their association with advanced clinical outcomes such as mortality or the need for intensive care are rare. In this regard, our study represents one of the few investigations aiming to explore the potential impact of salusins on clinical prognosis. Within this context, our findings suggest that salusins may have early diagnostic value but do not show a strong association with key prognostic indicators such as mortality or intensive care unit admission. This observation supports the notion that salusin peptides may act as early-phase inflammatory mediators in the pathogenesis of APE, yet their involvement in later-stage clinical processes—such as hemodynamic deterioration, right ventricular dysfunction, or tissue hypoxia—may be limited.

### 4.5. Limitations

Despite the novel contribution of this study, certain methodological limitations should be acknowledged as potential factors influencing the findings. First, the relatively small sample size may have reduced statistical power, especially in subgroup analyses. Additionally, the single-center design limits the generalizability of the results to broader clinical populations. The control group consisted of individuals presenting with non-specific symptoms and normal laboratory parameters, while patients with no evidence of embolism on CTPA measurements were excluded. Furthermore, salusin levels were measured only once at diagnosis, limiting the assessment of temporal changes and the dynamic role of these biomarkers during disease progression. The lack of long-term follow-up data prevented evaluation of the prognostic impact of salusin levels on advanced clinical outcomes. Moreover, patients with chronic pulmonary embolism were excluded from the present study; consequently, we could not assess the potential role of salusins in differentiating acute from chronic thromboembolic disease. This limitation is noteworthy, as distinguishing between acute and chronic cases is clinically important: a substantial residual thromboembolic burden in chronic cases predisposes patients to chronic thromboembolic pulmonary hypertension. Early identification of such cases may facilitate timely interventions—such as medical therapy, catheter-directed thrombolysis, or pulmonary endarterectomy—thereby potentially mitigating disease progression. Finally, potential confounding effects from cardiovascular and metabolic comorbidities could not be entirely excluded, which may have affected biomarker measurements.

### 4.6. Clinical Implications and Future Perspectives

Based on the present data, salusin-α appears to be a promising marker that could contribute to the diagnostic assessment of APE. Given the limited specificity of current diagnostic tools, the evaluation of salusin-α levels may contribute to clinical decision-making processes when combined with clinical assessments and laboratory findings. In patients who are clinically assessed as having a low probability of embolism but present with positive results in tests such as D-dimer, the use of salusin-α may help reduce unnecessary imaging procedures.

In contrast, the lack of a significant association between salusin-β levels and either thrombus burden or clinical risk scores suggests that this peptide may be more relevant to chronic inflammatory processes. To better understand the role of salusins throughout the course of the disease, there is a need for large-scale, multicenter studies involving measurements at different disease stages and time points. Such studies could clarify not only the diagnostic utility of salusins but also their potential impact on disease progression and long-term prognosis.

## 5. Conclusions

This study demonstrated that salusin-α exhibits strong sensitivity in the diagnosis of APE and may serve as a potential biomarker that could complement existing diagnostic approaches. Despite its high sensitivity of 89.5%, the limited specificity of salusin-α suggests that it may be particularly useful as a screening tool or as an indicator for the need for additional testing. In contrast, salusin-β levels did not show significant associations in multivariate analyses or in relation to risk scores. The findings indicate that salusin peptides may reflect an early biological response in APE but may not be sufficient for assessing disease severity or prognosis. Our results suggest that salusin-α, in particular, could be considered a helpful parameter for rapid diagnostic support in ED settings. Validation of these findings in larger cohorts and through longitudinal measurements will be essential to more clearly define the clinical utility of salusins.

## Figures and Tables

**Table 1:** Comparison of demographic, clinical, and laboratory characteristics between patients with acute pulmonary embolism and controls.

	APE (*n* = 57)	Control (*n* = 30)	*p*-Value
Age, years	61.96 ± 19.30	37.83 ± 8.35	<0.001
Gender, male, *n* (%)	29 (50.9%)	14 (46.7%)	0.709
BMI, kg/m^2^	26.7 (7.8)	24.6 (3.5)	<0.001
GCS	15 (0)	15 (0)	0.067
SBP, mmHg	118 (30)	125.5 (3)	0.014
DBP, mmHg	71 (12)	75 (4)	0.007
Respiratory rate, breaths/min	20 (8)	15(2)	<0.001
Heart rate, beats/min	90 (34)	76 (5)	<0.001
SpO_2_, %	91 (29)	95 (2)	<0.001
Body temperature, °C	36.5 (0.3)	36.5 (0.7)	0.406
Troponin, ng/L	14.80 (40.21)	3.12 (1.29)	<0.001
D-dimer, µg/mL	2.80 (3.77)	0.12 (0.09)	<0.001
BNP, pg/mL	365.00 (1406.15)	26.5 (24.53)	<0.001
Salusin-α, pg/mL	159.5 (62)	257.15 (477)	<0.001
Salusin-β, pg/mL	341.7 (390.55)	555.85 (600.70)	<0.001

Data are presented as the mean ± standard deviation or median (interquartile range), as appropriate. Abbreviations: APE, acute pulmonary embolism; BMI, body mass index; BNP, brain natriuretic peptide; DBP, diastolic blood pressure; GCS, Glasgow Coma Scale; SBP, systolic blood pressure; SpO_2_, peripheral oxygen saturation.

**Table 2:** Comparison of demographic, clinical, and laboratory characteristics between low-risk and high-risk patients with acute pulmonary embolism.

	Low Risk (*n* = 28)	High Risk (*n* = 29)	*p*-Value
Age, years	55.11 ± 20.41	68.59 ± 15.84	0.007
Gender, male, *n*	18 (64.3%)	11 (37.9%)	0.047
BMI, kg/m^2^	26.61 (12.38)	27.68 (6.46)	0.879
Comorbid diseases, *n*			
Diabetes mellitus	9 (32.1%)	9 (31.0%)	0.928
Hypertension	13 (46.4%)	16 (55.2)	0.509
COPD	8 (28.6%)	6 (20.7%)	0.490
CHF	5 (17.9%)	10 (34.5%)	0.154
CAD	7 (25.0%)	10 (34.5)	0.434
CVD	1 (3.6%)	2 (6.9%)	0.574
GCS	15 (0)	15 (0)	0.112
SBP, mmHg	120 (28)	114 (30)	0.216
DBP, mmHg	70.5 (12)	71.0 (16.0)	0.955
Respiratory rate, breaths/min	20 (9)	20 (8)	0.435
Heart rate, beats/min	88.5 (34.0)	96.0 (37.0)	0.170
SpO_2_, %	94.0 (7.0)	88.0 (10.0)	0.019
Body temperature, °C	36.5 (0.2)	36.5 (0.3)	0.656
Troponin, ng/L	8.23 (10.75)	39.40 (61.42)	0.001
D-dimer, µg/mL	1.50 (1.45)	4.08 (6.61)	<0.001
BNP, pg/mL	262.5 (694.38)	615.0 (2264.0)	0.079
Salusin-α, pg/mL	158.95 (73.00)	164.60 (56.00)	0.307
Salusin-β, pg/mL	333.50 (252.47)	355.20 (420.25)	0.854
PESI	84.14 ± 42.38	122.48 ± 47.44	0.002
Hospitalization period, day	5 (8)	8 (4)	0.106
ICU admission, *n*	3 (10.7%)	14 (48.3%)	0.002
Thrombolytic therapy, *n*	0 (0%)	6 (20.7%)	0.023
Mortality, *n*	1 (3.6%)	4 (13.8%)	0.352

Data are presented as the mean ± standard deviation or median (interquartile range), as appropriate. Risk classification was based on the PAOI: low-risk (≤20) and high-risk (>20). Abbreviations: BMI, body mass index; BNP, brain natriuretic peptide; CAD, coronary artery disease; CHF, congestive heart failure; COPD, chronic obstructive pulmonary disease; CVD, cerebrovascular disease; DBP, diastolic blood pressure; GCS, Glasgow Coma Scale; ICU, intensive care unit; PAOI, Pulmonary Artery Obstruction Index; PESI, Pulmonary Embolism Severity Index; SBP, systolic blood pressure; SpO_2_, peripheral oxygen saturation.

**Table 3:** Univariable and multivariable logistic regression analyses showing the association between clinical parameters and acute pulmonary embolism, including salusin-α.

	Univariable Regression		Multivariable Regression	
	OR (95% CI)	*p*-Value	OR (95% CI)	*p*-Value
Age	1.099 (1.054–1.147)	<0.001	1.113 (1.048–1.181)	<0.001
Gender, male	1.184 (0.488–2.870)	0.709	—	—
Body mass index	1.325 (1.124–1.563)	0.001	1.326 (1.042–1.686)	0.022
Systolic blood pressure	0.967 (0.937–0.997)	0.034	0.915 (0.845–0.992)	0.031
Salusin-α	0.996 (0.994–0.999)	0.030	0.995 (0.990–1.000)	0.042

Variables with *p* < 0.10 in the univariable analysis were entered into the multivariable model. Abbreviations: OR, odds ratio; CI, confidence interval; —, not included in the multivariable model.

**Table 4:** Univariable and multivariable logistic regression analyses showing the association between clinical parameters and acute pulmonary embolism, including salusin-β.

	Univariable Regression		Multivariable Regression	
	OR (95% CI)	*p*-Value	OR (95% CI)	*p*-Value
Age	1.099 (1.054–1.147)	<0.001	1.112 (1.050–1.178)	<0.001
Gender, male	1.184 (0.488–2.870)	0.709	—	—
Body mass index	1.325 (1.124–1.563)	0.001	1.284 (1.039–1.587)	0.021
Systolic blood pressure	0.967 (0.937–0.997)	0.034	0.918 (0.844–0.998)	0.045
Salusin-β	0.998 (0.996–0.999)	0.020	0.998 (0.996–1.000)	0.088

Abbreviations: OR, odds ratio; CI, confidence interval; —, not included in the multivariable model.

## Data Availability

Data utilized in this study are available upon reasonable request from the authors.

## Abbreviations

The following abbreviations are used in this manuscript:

APE	Acute pulmonary embolism
AUC	Area under the curve
CTPA	Computed tomography pulmonary angiography
ED	Emergency department
NT-proBNP	N-terminal pro-B-type natriuretic peptide
PAOI	Pulmonary Artery Obstruction Index
PESI	Pulmonary Embolism Severity Index
ROC	Receiver operating characteristic
SBP	Systolic blood pressure
Salusin-α	Salusin-alpha
Salusin-β	Salusin-beta
hs-TnT	High-sensitivity troponin T
